# Supportive Social Interactions in Infertility Treatment Decrease Cortisol Levels: Experimental Study Report

**DOI:** 10.3389/fpsyg.2019.02779

**Published:** 2019-12-13

**Authors:** Alicja Malina, Małgorzata Głogiewicz, Jakub Piotrowski

**Affiliations:** ^1^Department of Pedagogy and Psychology, Kazimierz Wielki University in Bydgoszcz, Bydgoszcz, Poland; ^2^Department of Obstetrics, Gynecology and Gynecological Oncology, Faculty of Health Sciences, Nicolaus Copernicus University in Toruń, Bydgoszcz, Poland; ^3^Department of Immunology, Faculty of Biology and Environment Protection, Nicolaus Copernicus University in Toruń, Torun, Poland

**Keywords:** *in vitro*, infertility and assisted reproductive techniques, social support, social interaction, cortisol

## Abstract

**Purpose:**

The aim of the research project was to analyze the importance of supportive social interactions in the process of infertility treatment. The acceptance rates of ART (Assisted Reproductive Technology) in Poland are lower than in western European countries and the social stigma of infertility exists. The research project draws attention to the issue of disclosure of fertility problems and the ability to seek support in Polish couples.

**Methods:**

An experimental study was conducted with 51 heterosexual couples who qualified for IVF. The participants were randomly divided into an experimental and control group. The first stage of the research procedure, with all the couples, was to extract a saliva (cortisol) sample as a biomarker for stress. In the second stage the control group viewed an informational (non-emotional) video about human embryology. The experimental group took part in a supportive social interaction process. In the supportive social interaction process, a maximum of five couples, were led through a broad general understanding of their IVF experience by an experienced group psychologist. The third stage of the research involved the second extraction of a saliva (cortisol) sample form all participants. In addition, demographic and medical history related to fertility was collected.

**Results:**

The statistical analysis indicates a significant decrease in the level of stress experienced after the supportive social interaction. The reported differences between the experimental group and the control group indicated a larger decrease of cortisol level for women and men.

**Conclusion:**

In the current study, the hypothesis that taking part in supportive social interaction significantly lowers stress levels (measured via cortisol) of infertile couples (men and women) was supported. Further the project indicates that a supportive social interaction has a beneficial effect on infertile couple’s health and well-being. The results of the study clearly point to the benefits of couples involved in infertility treatment to express and share their experience, and in doing so, provides measurable physiological and psychological benefits.

## Introduction

The acceptance and use of the Assisted Reproductive Technology (ART) has been systematically increasing over the last 30 years irrespective of the varying attitudes toward infertility treatment throughout the world (Kovacs et al., 2012). However, in Poland the acceptance of ART is still lower than in other Western European countries (Franklin, 2013) and the social stigma of infertility exists (Dembińska, Malina, in press). The varying rates of acceptance of ART depend on the religious and political context, and the financial and legal regulations within different countries. Despite the fact that *in vitro* fertilization (IVF) (one method of ART) has been available in Poland for 25 years, there is divided opinion regarding the legitimacy and the use of ART’s generally. The general public’s acceptance of the utilization of IVF by infertile couples has increased from 60% in 2008 to 76% in 2015^1^. Further, the acceptance rate for heterosexual couples using IVF, is 60%, compared to 44% for an individual. Despite the growing acceptance of IVF treatment in Poland, it is still much lower than in the developed Western European countries, where 93% of respondents support IVF being publicly funded. The acceptance rate of IVF is also reportedly much higher for families, for individuals (61%) and for same sex parents (64%) (Fauser et al., 2019). While the use of ART is slowly increasing in acceptance in Poland, the disclosure of the use of ART’s in general remains controversial. This may in part, explain the attitudes of general public toward IVF utilization (Dembińska and Malina, 2019). Whilst there are reported negative psychological effects of being infertile, the very process of ART and in particular IVF also impacts individuals (Eugster and Vingerhoets, 1999). Infertile partners tend to experience fear, sorrow, anxiety, distrust, and hostility (Csemiczky et al., 2000) and the experienced failures in conceiving a child are related to increased levels of depressions (Slade et al., 1997). Particular stages of the IVF process [everyday injections, blood testing, USG (Ultrasonography) examination, providing sperm samples etc.] may have an impact on the psycho-social functioning of the couple together and as individuals during this time of stress. Stress may also be related with the negative social appraisal of infertility treatment, as becoming a parent is an important part of adult social role and identity (Möller and Fällström, 1991; Malina et al., 2016). Different aspects of social assessment of the IVF procedure can intensify the feeling of loss, shame and social mismatch that often accompanies infertility (Whiteford and Gonzales, 1995; Monga et al., 2004; Pawelec and Pabian, 2012). The results of some studies indicate that the very process of infertility treatment tests the psychological adaptiveness of individuals and couples in a context where the inability to conceive a child overlaps with already existing somatic challenges (Conrad et al., 2002; Podolska and Bidzan, 2011).

It is noted that women and men react differently to infertility related stress (Benyamini et al., 2004, 2009). Not only are women more prone to the negative consequences of the stress related to the inability to carry a child, but it also impacts self-esteem and effect the evaluation of the marital relationship (Wright et al., 1991; Slade et al., 1997). Men, in turn, identify their infertility with sexual disability (Bidzan, 2010) which, may impact on self-esteem. Men report feelings of helplessness, worry about their infertility and guilt in relation to not fulfilling their partner’s needs (Glover et al., 1999). Men and women also use different coping strategies to deal with stress associated with infertility (Peterson et al., 2006).

Undoubtedly, stress is of particular importance when considering infertility issues, as the physiological processes associated with stress directly affects hormonal regulation, which can in turn affect the chances of pregnancy (Dembińska, 2012; Gourounti et al., 2012). Cortisol is a steroid hormone that is produced by the adrenal glands which when released into the bloodstream, acts on many different parts of the body, such as: increased metabolism of glucose, increased blood pressure or immune system suppression. Cortisol is best known for its involvement in the “fight-or-flight” response and temporary increase in energy production (Edwards, 2012; Whirledge and Cidlowski, 2013). However, elevated cortisol relating to prolonged stress can lend itself to erectile dysfunction or the disruption of normal ovulation and menstrual cycles. Furthermore, the androgenic sex hormones are produced in the same glands as cortisol, so excess cortisol production may hamper optimal production of these sex hormones (Weinstein, 2004). Stressful experiences and raised levels of cortisol appears to be implicated in the development of depression, a general decrease in psychological functioning and can contribute to the deterioration of somatic health (Richman, 2005). Importantly raised cortisol levels may also impact the chances of pregnancy (Galst, 2018), due to the immunological processes that are sensitive to the effects of emotions (Knapp, 1992). It is argued that positive emotions should outweigh the negative emotions three times to maintain mental and somatic health on a good level (Fredrickson, 2005).

This current research also draws attention to the issue of disclosure of fertility problems, which has been highlighted may be an issue for many polish couples. Disclosing may be a necessary involved in supportive social interactions. Access to supportive social interactions provides the opportunity for increasing self-esteem, mood, lowering stress (Domar et al., 1992; Dembińska, 2012), increasing general trust, confidence and a feeling of safety (Łuczak-Wawrzyniak and Pisarski, 1997) or assisting in increasing the quality of relationships (Perkins, 2006; Bączkowski et al., 2007; Ferraresi et al., 2013).

Studies indicate that social support reduces the levels of experienced stress in a range of contexts, infertility is one of these (Cobb, 1976; McNaughton-Cassill et al., 2000; Dudek and Koniarek, 2003; Giesbrecht et al., 2013; Koss et al., 2014; Ying et al., 2015a, b). One of the characteristics of infertility is the inability to use normal social support resources as the level of disclosure about infertility is low (Holas et al., 2002; Hoff et al., 2009). Therefore social support processes provide an first step to assist infertile couples dealing with infertility (Dembińska, 2012). Although there were many studies indicating the increase in subjective well-being of infertile couples due to psychological support, there have been few studies that have looked at social support and the impact on stress hormones level (Boivin, 2003).

Researchers looked at the meaning of social support in various circumstances such as:

– *Partner to partner support*: For both men and women, partner support was found to be negatively related to stress due to infertility. Partner support was an important element of coping with infertility (Ying et al., 2015a). Partner support significantly lowers negative emotions, pressure and worries (Koss et al., 2014). Nevertheless, studies showed that within the infertility context a partners support in many cases may not be sufficient due to the fact that both partners need for support. A high-quality, supportive partner relationship may also contribute to improved maternal and infant well-being postpartum, indicating a potential role for partner relationships in mental health interventions, with possible benefits for infants as well (Stapleton et al., 2012). Partner support may be an important and potentially modifiable target for interventions to improve pregnancy outcomes. Studies highlight higher levels of antenatal anxiety, depression, and smoking among pregnant women who report low partner support (Cheng et al., 2016). With the couples indicating that the support that they received from each other effected their experience during the treatment process, it is suggested that a supportive intervention that focuses on enhancing the partnership of the couples, and dealing with their inflexibility on the issue of bearing a child might result in improvements in the psychological status and marital relationship of infertile couples undergoing IVF treatment (Ying et al., 2015a).
– *Institutional support (individual or couple psychotherapy)*: couples report increased satisfaction with life, acceptance of own infertility and lower fear (Boivin, 2003; Martins et al., 2011; Mousavi et al., 2013). Psychological support has been suggested as reducing tension through relaxation training or behavioral treatment and improving conception rates (Eugster and Vingerhoets, 1999).
– *Informal support groups*: couples report feeling less stressed and point the importance of social bonds when being part of an informal support group (McNaughton-Cassill et al., 2000, 2002). At the same time the internet is changing people’s experience of infertility, giving people access to others’ experiences. The internet communication is highly valued by couples, especially those isolated in their real-world relationships (Hinton et al., 2010). According to some researchers’ infertility counseling and support groups seem to be the most efficient psychosocial interventions within the infertility context (Wischmann, 2008).

Couples admit that sharing emotions and supporting each other helps their well-being and creates better partnership (Ying et al., 2015b). Supportive social interactions are crucial factors in coping with stressful situation of infertility treatment. In Poland where, the acceptance of ART is still relatively low, supportive social interactions are not a natural choice for many couples who struggle with disclosing issues about fertility to friends or family. The research on supportive social interactions indicating an effect on stress symptoms and stress hormones offers an opportunity to examine this process empirically.

The aim of the research project was to analyze the importance of supporting social interactions in the process of reducing stress during infertility(IVF) treatment. Supportive social interactions include sharing experiences, psychological needs or personal beliefs of people participating in this interaction. Therefore, results of “getting” or “receiving” psychological assistance in supportive social interactions should include: achieving acceptance of own limitations, greater sense of security and mental comfort, increased motivation or readiness to take action. We define supportive social interaction as *a group interaction involving talking or listening in an informal and non-judgmental environment, which results in stress reduction.* The authors hypothesize that supportive social interactions in the context of infertility treatment will alter infertility related stress (Eugster and Vingerhoets, 1999; Boivin, 2003; Martins et al., 2011; Mousavi et al., 2013).

The main hypothesis of the study was: Taking part in supportive social interaction significantly lowers stress level measured with biomarkers (cortisol) of infertile couples (men and women).

## Materials and Methods

The design of the study was experimental. 51 heterosexual couples were recruited for the study by a gynecologist. A systematic sampling was conducted until achieving the total planned sample size determined by the funding conditions. The selection criteria was ART qualification. All volunteers reported to be in good health and had no history of mental disorders. The mean age of all participants was 32 years (SD = 4.20, min = 23, max = 43). The mean age of female participants was 31 (SD = 3.9). The mean age of male participants was 33 (SD = 4.7). Almost all couples were married (45 couples), others claimed they were engaged (6 couples) for the average time of 9 years (SD = 4.20, min = 3, max = 20). And being diagnosed as infertile for an average of 39 months (SD = 33 min = 1, max = 180). Twenty-one couples declined to participate in the study. Only four couples that declared participation in the experimental study dropped out. All the couples were recruited in infertility treatment clinics in five Polish cities in the period from May 2018 to January 2019.

The majority of couples were qualified for their first *in vitro* procedure (41) after unsuccessful intrauterine insemination, four couples had one transfer before, four had two transfers, and one couple taking part in the study had three IVF transfers before. Five couples reported to already have children from previous procedures or other relationships. The full information about the descriptive statistics is presented in the Tables 1, 2.

**TABLE 1:** Descriptive statistics for quantitative variables in the studied sample.

**Variable**	**Min**	**Max**	**M**	**SD**
Age (years)	23	43	32.20	4.20
Relationship length (years)	3	20	9.02	4.20
Time since diagnosis (months)	1	180	38.91	33.23
Number of IVF	0	4	1	0.98
Which insemination	0	4	1	1.40

*Source: own data.*

**TABLE 2:** Descriptive statistics for quantitative variables in the studied sample.

***n* = 102**	**Frequency**	**Percent**
**Relationship**		
Married	90	88.2
Engaged	12	11.8
**Infertility reason**		
Primary	48	47.1
Secondary	32	31.4
Unknown	22	21.5
**Infertitlite partner**		
Both	22	21.6
Female	42	41.2
Male	10	9.8
Idiopatic	28	27.4
**Using gamet donor**		
Yes	6	5.0
No	94	95.0
**Having children**		
Yes	12	11.8
No	90	88.2
**Who is aware of the issue?**		
Everyone	16	15.7
No one	14	13.7
Family	30	29.4
Family and friends	42	41.2
**Who is the source of support**		
Partner only	70	68.6
Family and friends	10	9.8
Family	16	15.7
No one	6	5.9
**Institutional support**		
Yes	12	11.0
No	92	89.0

*Source: own data.*

All the couples taking part in the experimental study were qualified for ART according to the recommendations of the Polish Society of Reproductive Medicine and Embryology (PTMRiE) and the Polish Society of Gynecologists and Obstetricians (PTGP)^2^. All the couples have not conceived after one year of unprotected vaginal sexual intercourse in the absence of any known cause of infertility. In all 51 cases the diagnostics of fertility causes was carried out in both partners. The aim was to determine the causes of childlessness and to develop individualized treatment. In the first place the diagnostics included monitoring of the ovarian function, female reproductive system anatomy and semen analysis, hormonal and biochemical tests (Pfeifer et al., 2015). The couples started with 6 months of ovarian stimulation (clomifene citrate/letrozole/metformin or a combination of the above) (Roque et al., 2015; Tatsumi et al., 2017) with the ultrasound monitoring during the cycles of treatment and timed sexual intercourse. All the couples who have not conceived within six cycles were qualified for 3–6 cycles of intrauterine insemination (IUI) (Farquhar et al., 2018) and in the end who have not conceived during IUI cycles to *in vitro* fertilization. Where investigations show there is no chance of pregnancy with expectant management and where *in vitro* fertilization is the only effective treatment, the woman was directly refereed to a specialist team for the *in vitro* treatment.

Before recruiting the participants, an approval of Bioethical Committee of Nicolaus Copernicus University was obtained^3^. The study was conducted in five subgroups of 10–11 couples. Therefore, a total number of couples taking part in the study was 51 couples: 26 in experimental and 25 in control group. The experiment was conducted always with the use of three separate rooms on Saturday morning. The whole procedure took 6–7 h. Before the start of the experiment couples had to wait 10–15 min to enter, they did not know each other and had no specific information about what was going to happen. This enabled them time to focus on the experimental/control tasks and relate it to their current medical situation. Before the data collection, participants were asked to sit down in room 1, given an informed consent form and general information about the procedure and their rights. To mask the real aim of the study the participants were told that the study concerns psychological aspects of infertility. Participants were then randomly allocated between the experimental and control group by drawing a number. The study was fully anonymous.

The first stage of the research procedure was carried out with participation of couples from both groups. It included taking a saliva sample to obtain information about the level of stress based on the cortisol test. The couple was taken to a separate room (room 2) for their comfort. Saliva was collected from a voluntaries into pure polypropylene tubes. As food might contain steroid hormones, samples were taken while fasting. Saliva flow was stimulated only by drinking water, but drinking was not allowed during the last 5 min before taking samples.

As the cortisol secretion in saliva shows an obvious pattern through the day and there might be smaller peaks in the secretion, three separate samples were collected within an hour before and after experiment. We also asked a simple question to record subjective stress for base line. The question was: “On a scale on 1–10 how stressed are you at the moment (where 1 means not stressed at all and 10 means extremely stressed).”

In the second stage of the experiment (right after collecting samples from all participants) couples were taken back to room 1 (control group) or room 3 (experimental group). The control group watched a non-emotional 150 min video about human embryology. The couples watched the film as a group but did not have the opportunity to make comments or communicate. At the same time the experimental group was the subject to a supportive social interaction. The interaction was conducted in a group of 5–6 couples and was a conversation about couples hopes and fears. There was a psychologist in the room to moderate the discussion but he did not get involved it the conversation itself. The supportive social interaction lasted between 3 and 5 h depending on the need and will of the participants. The participants were encouraged but not forced to speak. They spoke one at the time spontaneously. The conversation was fully led by their needs and had no planned structure. At the beginning of the interaction the psychologist presented rules (e.g., We don’t judge, We don’t interrupt) and asked an auxiliary question concerning their feeling regarding infertility treatment. In all five groups all the participants spoke with a different frequency – some wanted to say more and some were rather quiet and listened to what others had to say.

After introducing the experimental and control condition a saliva sample was again collected (third stage) from all participants in the same pattern as mentioned before. The information about the history of infertility treatment was collected.

After the experiment all the couples were debriefed (control and experimental group separately to avoid long waiting) and full information about the aim of the study was given. Saliva samples were mailed in cooling box (2–8°C) to the laboratory. Upon arrival samples were frozen (−20°C) overnight. Before the assay the frozen samples were warmed to room temperature and mixed carefully. Then centrifuged 1000 × *g* for 5 min. Clear colorless supernatants were collected, and reddish samples were discarded. Cortisol levels in samples was determined using solid phase enzyme-linked immunosorbent assay (ELISA) diagnostic kits from Demeditec Diagnostics GmbH (Kiel, Germany; cat. no. DES6611) with detection limits of 0.1 ng/ml. Colorimetric changes were detected using a Synergy HT Multi-Mode Microplate Reader (BioTek Instruments, United States). All samples were assayed individually in duplicates.

Statistical analyses were performed with GraphPad Prism 7.00 (GraphPad Software Inc., San Diego, CA, United States).

Only disposable and sterile tubes were used for saliva collection. Researchers wore gloves while collecting saliva samples, and at all times while handling saliva or materials used to collect saliva. Unused saliva as well as all materials used for experiments (tubes, gloves, pipette tips, etc.) were sterilized and disposed of as biohazardous material to prevent transmission of potentially infectious materials from participants.

## Results

Results from 86 participants (45 individuals in the experimental group and 41 individuals in the control group) were recorded during cortisol level assessment. False positive (highly above the expected normal) values results were rejected.

To determine if taking part in supportive social interaction significantly lowers stress levels measured with biomarkers (cortisol) of infertile couples (men and women) a comparison of mean decrease in cortisol level (delta) in experimental and control group was performed. First, analysis of the delta distribution (difference in cortisol level after the study and before the test) was performed in the experimental group and in the control group. The analysis indicates that the distributions for the delta variable in the experimental group (*W* = 0.95, *p* > 0.05) and in the control group (*W* = 0.96, *p* > 0.05) are similar to normal distribution. Two-way ANOVA followed by a Bonferroni pairwise comparison was used to test for statistical differences among groups. Differences were considered significant at *P* < 0.05.

To determine the relationship between the subjective stress perception (based on questionnaire survey) in different groups and their relationship with objective stress (saliva cortisol concentration) a multivariate discriminant analysis (Canonical Variate Analysis; CVA) was performed. Statistical analyses were performed with Past 3.25. Simultaneously, with the CVA a Monte Carlo permutation test was used to evaluate the statistical significance of the correlations and differentiate between experimental groups.

**FIGURE 1:**
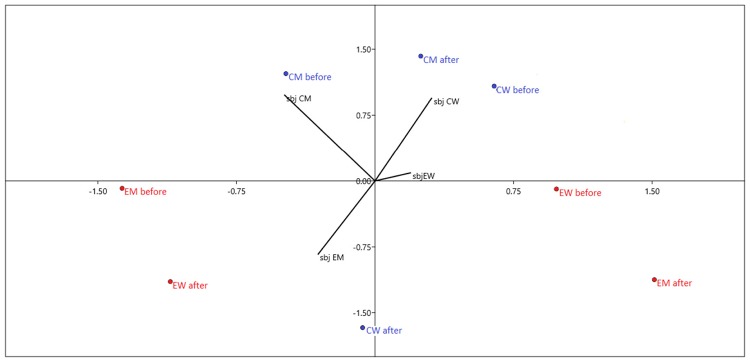
Canonical variate analysis (CVA) diagram relating subjective stress perception (lines) in different groups to objective stress measured as saliva cortisol concentration (dots). The greater the length of the line, the greater its importance. The closer the line to the dots, the greater their correlation. EW, experimental group of women; CW, control group of women; EM, experimental group of men; CM, control group of men; sbj, subjective stress perception (scale 1–10); “before,” “after” – saliva cortisol level before and after supportive social interaction or control treatment, respectively.

The ordination diagram, Figure 1, emphasizes the link between the levels of the examined parameters. It shows a strong correlation between the subjective stress perception and saliva cortisol concentration in women (*r* = 0.54) and men (*r* = 0.65) in control group before the control treatment. The relationship between subjective stress perception and cortisol levels in both, women and men experimental groups before supportive social interaction has not been statistically significant.

Mean decrease in saliva cortisol concentration was calculated as difference between cortisol concentration before and after the supportive social interaction in each individual volunteer. Values are expressed as means ± SEM. False positive (highly above the expected normal) values were rejected, therefore the graph demonstrates results for 23 women in experimental group and 20 in control group and 23 men in experimental group and 21 in control group.

**FIGURE 2:**
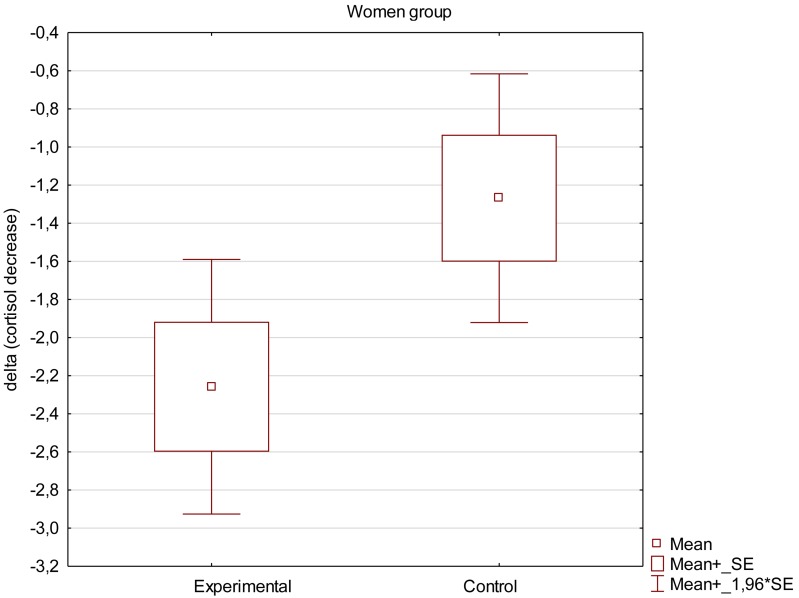
Mean decrease in saliva cortisol concentration in women.

**FIGURE 3:**
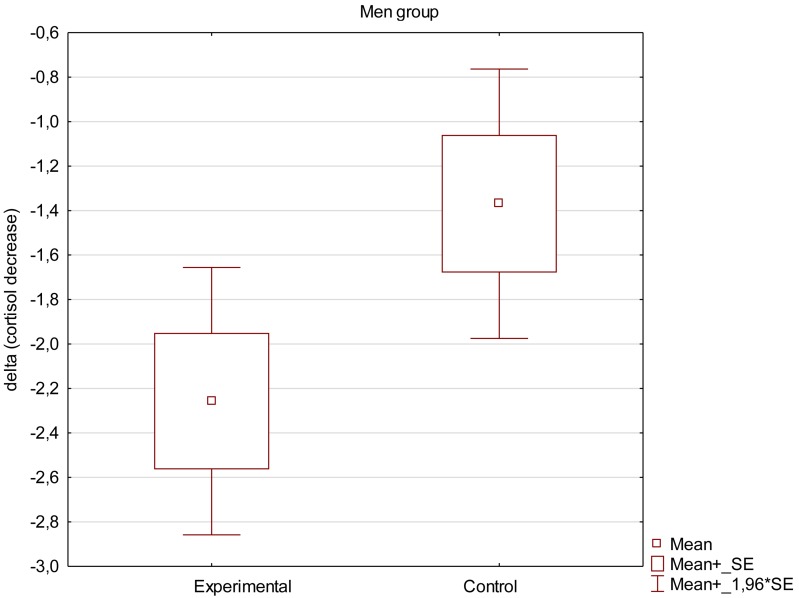
Mean decrease in saliva cortisol concentration in men.

Obtained results demonstrate that the decrease in saliva cortisol concentration was higher in experimental groups than in control groups, both in women and men as can be seen in Figures 2, 3. The mean decrease observed in women was slightly higher (−2.26 ± 0.32 ng/ml in comparison to −1.27 ± 0.3 ng/ml in control group; *p* = 0.043) than in men (−2.26 ± 0.29 ng/ml in experimental group compared to −1.36 ± 0.28 ng/ml in control group, respectively; *p* = 0.045). The results are presented in Table 3. Examing the impact of supportive social interaction on both sexes separately should be the subject of in-depth analysis in subsequent works due to the fact that results suggest that both sexes may react differently to the interaction.

**TABLE 3:** Descriptive statistics for quantitative variables in the studied sample.

	**Subjective stress perception (scale 1–10)**					**Saliva cortisol concentration before supportive social interaction or control treatment (ng/ml)**				**Saliva cortisol concentration after supportive social interaction or control treatment (ng/ml)**			
			
	***N***	**Mean**	**SEM**	**q 1-3**	***p***	**Mean**	**SEM**	**q 1–3**	***P***	**Mean**	**SEM**	**q 1–3**	***P***
Experimental women group	22	5.045	0.33	4.75–6		5.60	0.47	3.75–7.72		3.35	0.36	2.35–3.32	
Control women group	20	4	0.61	1.5–6	0.83	3.52	0.41	1.98–4.79	0.14	2.25	0.20	1.39–3.03	0.04^∗^
Experimental men group	23	3.73	0.49	2–5		5.62	0.56	3.32–6.07		3.36	0.37	1.93–4.45	
Control men group	20	3.71	0.50	2–5	0.99	3.96	0.42	1.84–5.05	0.085	2.59	0.27	1.37–3.52	0.048^∗^

*Asterisks indicate significant difference between control and experimental group (^∗^*p* < 0.05). The results were analyzed using two-way ANOVA followed by a Bonferroni pairwise comparison test.*

## Discussion

The current study sought to test the hypothesis taking part in supportive social interaction significantly lowers stress level measured with biomarkers (cortisol) of infertile couples (men and women).

Due to high effectiveness of the ART methods, they are popular. Nevertheless, these methods are associated with a large interference in the intimacy of the treated couple and so extremely difficult from the psychological perspective. Various aspects of the negative social appraisal of assisted reproductive technology present in the Polish society may intensify the feeling of loss, shame and social maladjustment which frequently accompany infertility (Whiteford and Gonzales, 1995; Monga et al., 2004; Pawelec and Pabian, 2012; Dembińska, 2018). There is a widely held conviction that a hindered trying for a baby is a stressful factor which may influence the early behavior of parents, hence it may potentially influence the psychosocial development of a child and the whole family system (Hahn and DiPietro, 2001; MacCallum et al., 2007). Therefore, it is worthwhile to pay attention to the importance of the perception of social attitudes both with regards to using assisted reproductive technology and revealing information about the method of conception which may be a significant factor when seeking for social support.

The presented study reflects the meaning of supportive social interactions in infertility treatment. Previous research in the field of social support provided for infertile couples was focused on perceived support of closest family or friends or different institutional forms of support (McNaughton-Cassill et al., 2000, 2002; Boivin, 2003; Martins et al., 2011; Mousavi et al., 2013; Koss et al., 2014). In this project the authors intention was to pinpoint that a supportive social interaction is an interaction where a couple is able to share their experiences in a safe, non-judgmental environment that does not have to be in any specific form or does not have to involve a specialist, e.g., therapist. To interpret the different results obtained in subgroups, it is worth mentioning previous studies indicating that for women it is more difficult to deal with the diagnosis and process of infertility treatment than to men (McEwan et al., 1987; Ulbrich et al., 1990; Berg and Wilson, 1991; Benazon et al., 1992; Gibson and Myers, 2002). Both men and women are satisfied with medical care in the treatment of infertility (Schmidt, 2006); nevertheless, men and women use different coping strategies to deal with stress associated with infertility (Peterson et al., 2006). Women tend to confront, take responsibility, seek social support, while men exhibit techniques of distancing themselves, self-control and planned problem solving (Peterson et al., 2006), which could be an explanation of why women benefit more from a supportive social interaction than their partners. Also, studies indicate that men prefer to receive emotional support from infertility clinicians rather than from mental health professionals, self-help support groups or friends. Nevertheless, structured, facilitated psycho-educational groups that are didactic but permit informal sharing of experiences might be beneficial (Fisher and Hammarberg, 2012). Unlike women, men are not eager to talk about their feelings connected with infertility which may hinder receiving social support (Bielawska-Batorowicz, 1991). Studies highlight the importance of social support contexts in helping to deal with infertility treatment (Martins et al., 2013). Suggesting that health professionals should explore the quality of social networks and encourage seeking positive support from family and partners (Martins et al., 2011). On the other hand, the lack of social support constitutes an important risk factor for maternal well-being during pregnancy and has adverse effects on pregnancy outcomes (Elsenbruch et al., 2007).

It is difficult to find results that directly relate to the present study. However, the effects of short-term social support on cortisol levels was investigated before in healthy adults. For instance, a study by Kirschbaum et al. (1995) in anticipation of a public-speaking task subjects received either no social support or social support from an opposite-sex stranger or from their boyfriend or girlfriend. The results obtained also suggest sex-specific effects of social support. Although men in the partner support condition showed significant attenuation of cortisol responses compared with unsupported and stranger-supported men, women showed no response decrement under stranger support. In contrast to men, women showed a tendency toward increased cortisol responses when supported by their boyfriends. In a different study by Giesbrecht et al. (2013). The buffeting effect of social support on hypothalamic-pituitary-adrenalaxis function during pregnancy was tested. The results indicate that pregnant women receiving inadequate social support secrete higher levels of cortisol in response to psychological distress as compared with women receiving effective social support. Both of the above mentioned studies suggest that the obtained decrease in cortisol level among infertile women might be not only due to a supportive social interaction with other couples but also the feeling of being supported by the partner.

Research conducted by Ditzen et al. (2019) supports the explanation. The study investigated whether the couples’ spontaneous expression of intimacy before and after psychosocial stress exposure in the laboratory reduced cortisol reactivity and accelerated recovery. Data from 183 couples (366 individuals) were analyzed. Obtained results indicate that observed partner intimacy reduced cortisol responses to stress in women. Spontaneous non-verbal expressions of intimacy regulate the effects of acute environmental demands on established biological indices of stress response. Undoubtedly, the couples taking part in the experimental task had an opportunity to express such intimacy during the supportive social interaction. It can be presumed that sharing intimate information is itself an indicator of intimacy.

There has not been reports of the actual effect of support in infertility made with biomarkers. As mentioned above previous studies concerning social support were made with the use of self-report questionnaires or interviews. The obtained results were more reliable and it demonstrates that the decrease in saliva cortisol concentration was higher in experimental group than in control group, both in women and men. This means that couples taking part in the supportive social interaction experienced a decrease of the level of emotional tension (stress) operationalized as the level of cortisol in saliva sample. Mean decrease observed in women was slightly higher than in men.

We find the study an important source of information on how couples going through *in vitro* fertilization cope with accompanying stress. The results indicate the legitimacy of monitoring objective stress (cortisol concentration) in biological material collected in a stress-free environment when assessing the effectiveness of social support.

Furthermore, in the project the authors intention was to pinpoint that a supportive social interaction has a beneficial effect on couple’s health and well-being. This is an important implication especially in the Polish society where sharing the sensitive information on fertility issues has been proven to be difficult for many couples (Dembińska, 2018; Dembińska and Malina, 2019). The research shows that support as a feeling of acceptance and understanding may be due to contact with professionals (psychologists) and non-professionals (friends, family members, other infertile couples). Information about the access to such support should be collected by the gynecologist and sufficient sources of support should be provided by hospitals. The results of the study can be useful when preparing psychoeducation material for those couples.

The results of the study clearly indicate the measurable benefits of the psychologist’s work with couples undergoing or preparing for *in vitro* fertilization. Nevertheless, the study indicates the necessity of conducting further research aimed to correlate the results of the assessment of the effectiveness of the support procedure, the level of objective stress and the further success of the *in vitro* procedure. Extremely valuable from a cognitive point of view should be deepened (requiring a much larger test sample) linking the objective stress level (and other biological parameters) with the reported stress level (subjective) and many other variables, such as gender, age, relationship status.

The presented research, despite significant scientific values, also has some limitations. One of them concerns the limited possibility of generalizing research results. The manuscript describes preliminary studies that prove the need for further in-depth analysis of the issue on a larger sample.

Also, from a methodological point of view, rigorous approach to preparing patients for sampling turned out to be extremely important. It indicates the need to develop better procedures that preclude obtaining falsified results for future research. It would also be valuable to control variables such as perceived stress level before and after the experimental condition and whether participants perceived the experimental condition as actually supportive. From the medical perspective it would also be worth controlling the menstrual cycle phase and pharmacological supplementation. The time spent by the participants under the experimental and control condition should also be taken under consideration when interpreting the results. Although in most cases the duration of the supportive social interaction was 3 h, since the intervention was led by the participants it did last 5 h in case of one group. This could be a possible threat to internal validity as the control condition (exposition to a film) lasted less than 3 h.

## Data Availability Statement

The datasets generated for this study are available on request to the corresponding author.

## Ethics Statement

This study was carried out in accordance with the recommendations of Polish Psychological Association Ethical Guidelines with written informed consent from all subjects. All subjects gave written informed consent in accordance with the Declaration of Helsinki. The protocol was approved by the Collegium Medicum UMK Bioethical Committee.

## Author Contributions

AM contributed to the conception and design of the study and organized the database. JP performed the statistical analysis and wrote the section “Results” of the manuscript. MG contributed to the theoretical background. AM, JP, and MG contributed to the manuscript revision, and read and approved the submitted version.

## Conflict of Interest

The authors declare that the research was conducted in the absence of any commercial or financial relationships that could be construed as a potential conflict of interest.

## Footnotes

1 https://cbos.pl/SPISKOM.POL/2015/K_096_15.PDF (accessed July 15, 2019).

2 http://ptmrie.org.pl/akty-prawne-i-rekomendacje-art/rekomendacje/ (accessed July 15, 2019).

3 Approval numer: KB343/2018; dated: 24.04.2018.
